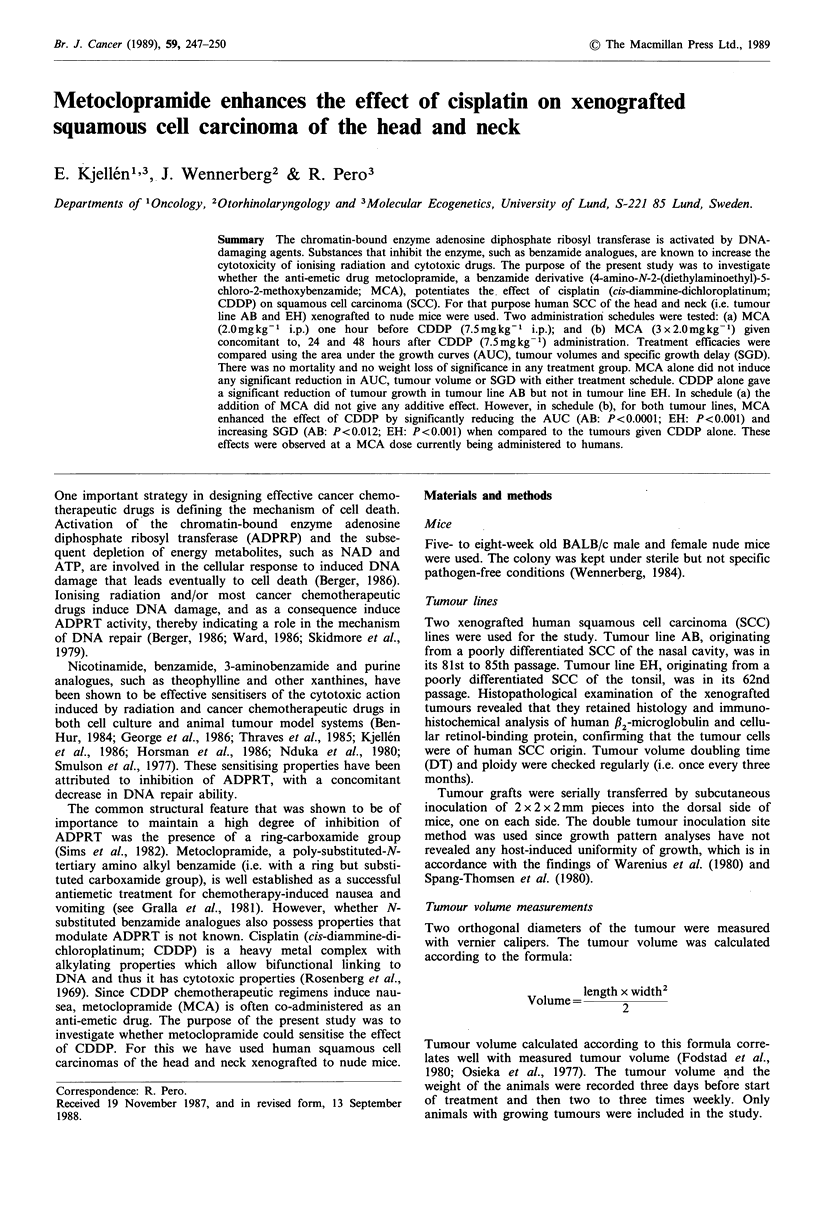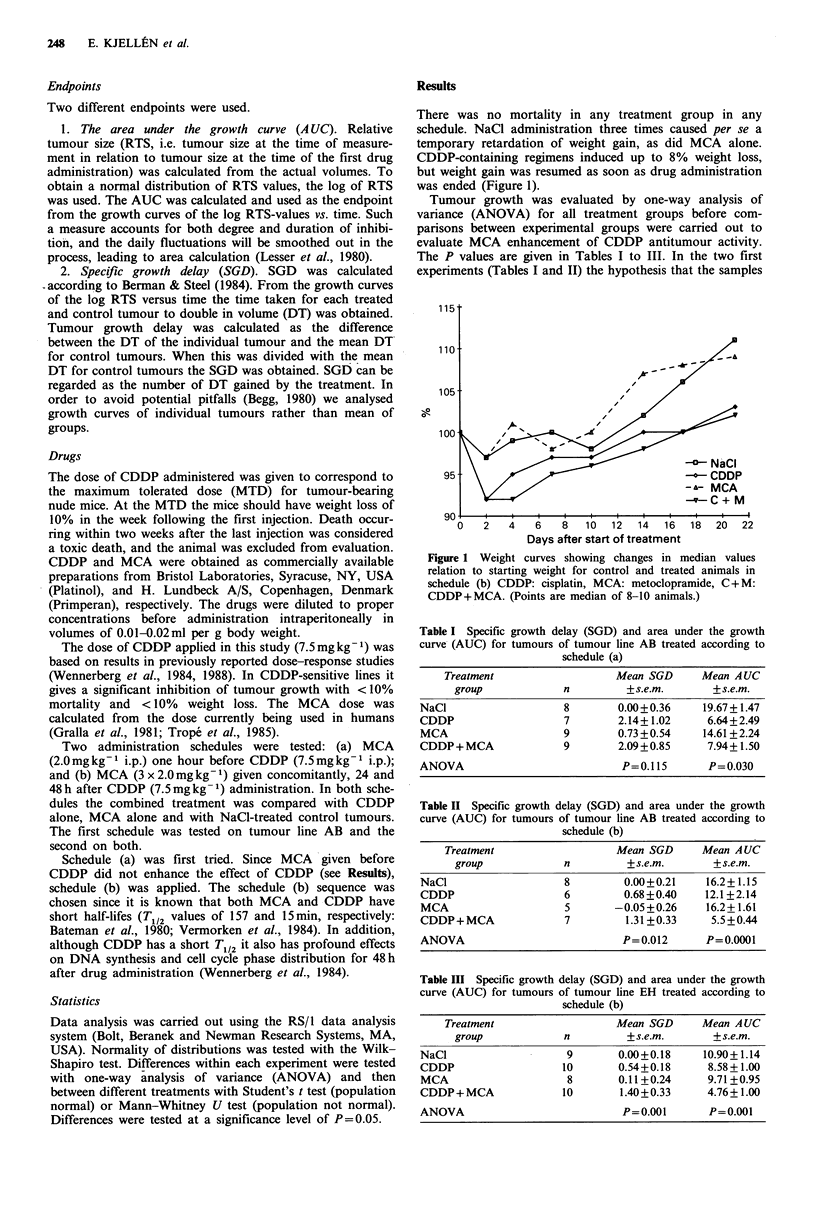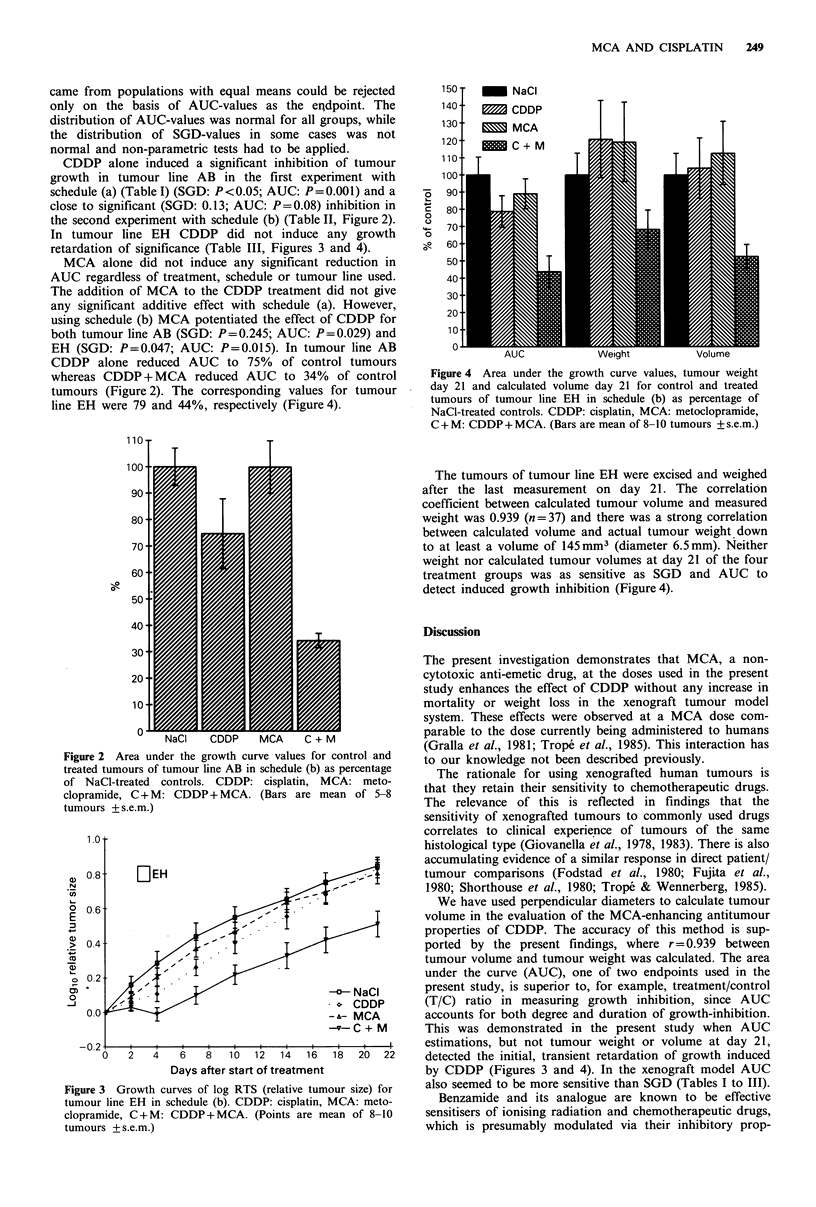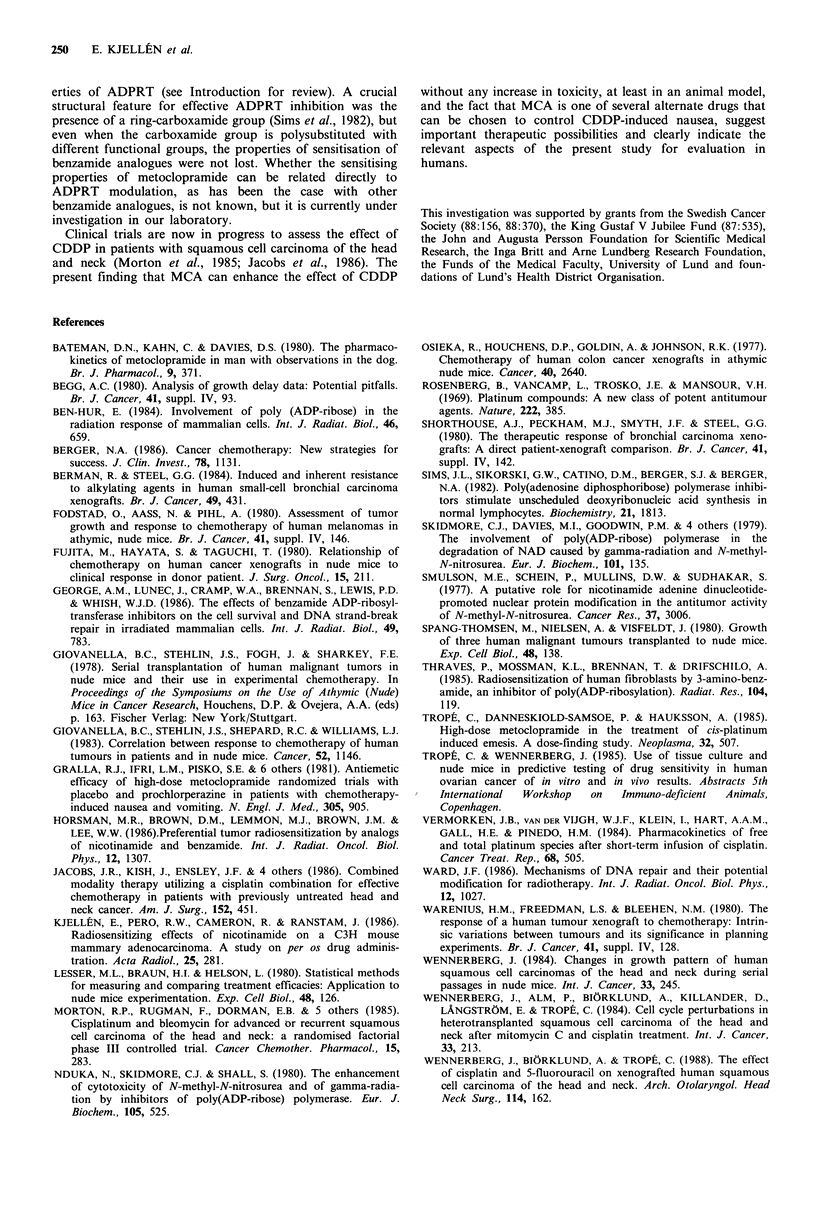# Metoclopramide enhances the effect of cisplatin on xenografted squamous cell carcinoma of the head and neck.

**DOI:** 10.1038/bjc.1989.50

**Published:** 1989-02

**Authors:** E. KjellÃ©n, J. Wennerberg, R. Pero

**Affiliations:** Department of Oncology, University of Lund, Sweden.

## Abstract

The chromatin-bound enzyme adenosine diphosphate ribosyl transferase is activated by DNA-damaging agents. Substances that inhibit the enzyme, such as benzamide analogues, are known to increase the cytotoxicity of ionising radiation and cytotoxic drugs. The purpose of the present study was to investigate whether the anti-emetic drug metoclopramide, a benzamide derivative (4-amino-N-2-(diethylaminoethyl)-5-chloro-2-methoxybenzamide; MCA), potentiates the effect of cisplatin (cis-diammine-dichloroplatinum; CDDP) on squamous cell carcinoma (SCC). For that purpose human SCC of the head and neck (i.e. tumour line AB and EH) xenografted to nude mice were used. Two administration schedules were tested: (a) MCA (2.0 mg kg-1 i.p.) one hour before CDDP (7.5 mg kg-1 i.p.); and (b) MCA (3 x 2.0 mg kg-1) given concomitant to, 24 and 48 hours after CDDP (7.5 mg kg-1) administration. Treatment efficacies were compared using the area under the growth curves (AUC), tumour volumes and specific growth delay (SGD). There was no mortality and no weight loss of significance in any treatment group. MCA alone did not induce any significant reduction in AUC, tumour volume or SGD with either treatment schedule. CDDP alone gave a significant reduction of tumour growth in tumour line AB but not in tumour line EH. In schedule (a) the addition of MCA did not give any additive effect. However, in schedule (b), for both tumour lines, MCA enhanced the effect of CDDP by significantly reducing the AUC (AB: P less than 0.0001; EH: P less than 0.001) and increasing SGD (AB: P less than 0.012; EH: P less than 0.001) when compared to the tumours given CDDP alone. These effects were observed at a MCA dose currently being administered to humans.


*Br. J. Cancer* (1989), **59**, 247–250 © The Macmillan Press Ltd., 1989

### Metoclopramide enhances the effect of cisplatin on xenografted squamous cell carcinoma of the head and neck

E. Kjellén[1,3], J. Wennerberg[2] & R. Pero[3]

*Departments of [1]Oncology, [2]Otorhinolaryngology and [3]Molecular Ecogenetics, University of Lund, S-221 85 Lund, Sweden.*

**Summary** The chromatin-bound enzyme adenosine diphosphate ribosyl transferase is activated by DNA-damaging agents. Substances that inhibit the enzyme, such as benzamide analogues, are known to increase the cytotoxicity of ionising radiation and cytotoxic drugs. The purpose of the present study was to investigate whether the anti-emetic drug metoclopramide, a benzamide derivative (4-amino-*N*-2-(diethylaminoethyl)-5-chloro-2-methoxybenzamide; MCA), potentiates the effect of cisplatin (*cis*-diammine-dichloroplatinum; CDDP) on squamous cell carcinoma (SCC). For that purpose human SCC of the head and neck (i.e. tumour line AB and EH) xenografted to nude mice were used. Two administration schedules were tested: (a) MCA ($2.0\,mg\,kg^{-1}$ i.p.) one hour before CDDP ($7.5\,mg\,kg^{-1}$ i.p.); and (b) MCA ($3\times2.0\,mg\,kg^{-1}$) given concomitant to, 24 and 48 hours after CDDP ($7.5\,mg\,kg^{-1}$) administration. Treatment efficacies were compared using the area under the growth curves (AUC), tumour volumes and specific growth delay (SGD). There was no mortality and no weight loss of significance in any treatment group. MCA alone did not induce any significant reduction in AUC, tumour volume or SGD with either treatment schedule. CDDP alone gave a significant reduction of tumour growth in tumour line AB but not in tumour line EH. In schedule (a) the addition of MCA did not give any additive effect. However, in schedule (b), for both tumour lines, MCA enhanced the effect of CDDP by significantly reducing the AUC (AB: $P<0.0001$; EH: $P<0.001$) and increasing SGD (AB: $P<0.012$; EH: $P<0.001$) when compared to the tumours given CDDP alone. These effects were observed at a MCA dose currently being administered to humans.

One important strategy in designing effective cancer chemotherapeutic drugs is defining the mechanism of cell death. Activation of the chromatin-bound enzyme adenosine diphosphate ribosyl transferase (ADPRP) and the subsequent depletion of energy metabolites, such as NAD and ATP, are involved in the cellular response to induced DNA damage that leads eventually to cell death (Berger, 1986). Ionising radiation and/or most cancer chemotherapeutic drugs induce DNA damage, and as a consequence induce ADPRT activity, thereby indicating a role in the mechanism of DNA repair (Berger, 1986; Ward, 1986; Skidmore *et al.*, 1979).

Nicotinamide, benzamide, 3-aminobenzamide and purine analogues, such as theophylline and other xanthines, have been shown to be effective sensitisers of the cytotoxic action induced by radiation and cancer chemotherapeutic drugs in both cell culture and animal tumour model systems (Ben-Hur, 1984; George *et al.*, 1986; Thraves *et al.*, 1985; Kjellén *et al.*, 1986; Horsman *et al.*, 1986; Nduka *et al.*, 1980; Smulson *et al.*, 1977). These sensitising properties have been attributed to inhibition of ADPRT, with a concomitant decrease in DNA repair ability.

The common structural feature that was shown to be of importance to maintain a high degree of inhibition of ADPRT was the presence of a ring-carboxamide group (Sims *et al.*, 1982). Metoclopramide, a poly-substituted-*N*-tertiary amino alkyl benzamide (i.e. with a ring but substituted carboxamide group), is well established as a successful antiemetic treatment for chemotherapy-induced nausea and vomiting (see Gralla *et al.*, 1981). However, whether *N*-substituted benzamide analogues also possess properties that modulate ADPRT is not known. Cisplatin (*cis*-diammine-dichloroplatinum; CDDP) is a heavy metal complex with alkylating properties which allow bifunctional linking to DNA and thus it has cytotoxic properties (Rosenberg *et al.*, 1969). Since CDDP chemotherapeutic regimens induce nausea, metoclopramide (MCA) is often co-administered as an anti-emetic drug. The purpose of the present study was to investigate whether metoclopramide could sensitise the effect of CDDP. For this we have used human squamous cell carcinomas of the head and neck xenografted to nude mice.

Correspondence: R. Pero.
Received 19 November 1987, and in revised form, 13 September 1988.

### Materials and methods

### *Mice*

Five- to eight-week old BALB/c male and female nude mice were used. The colony was kept under sterile but not specific pathogen-free conditions (Wennerberg, 1984).

### *Tumour lines*

Two xenografted human squamous cell carcinoma (SCC) lines were used for the study. Tumour line AB, originating from a poorly differentiated SCC of the nasal cavity, was in its 81st to 85th passage. Tumour line EH, originating from a poorly differentiated SCC of the tonsil, was in its 62nd passage. Histopathological examination of the xenografted tumours revealed that they retained histology and immunohistochemical analysis of human $\beta_2$-microglobulin and cellular retinol-binding protein, confirming that the tumour cells were of human SCC origin. Tumour volume doubling time (DT) and ploidy were checked regularly (i.e. once every three months).

Tumour grafts were serially transferred by subcutaneous inoculation of $2\times2\times2\,mm$ pieces into the dorsal side of mice, one on each side. The double tumour inoculation site method was used since growth pattern analyses have not revealed any host-induced uniformity of growth, which is in accordance with the findings of Warenius *et al.* (1980) and Spang-Thomsen *et al.* (1980).

### *Tumour volume measurements*

Two orthogonal diameters of the tumour were measured with vernier calipers. The tumour volume was calculated according to the formula:

$$\text{Volume} = \frac{\text{length}\times\text{width}^2}{2}$$

Tumour volume calculated according to this formula correlates well with measured tumour volume (Fodstad *et al.*, 1980; Osieka *et al.*, 1977). The tumour volume and the weight of the animals were recorded three days before start of treatment and then two to three times weekly. Only animals with growing tumours were included in the study.

*Endpoints*

Two different endpoints were used.

1. *The area under the growth curve* (*AUC*). Relative tumour size (RTS, i.e. tumour size at the time of measurement in relation to tumour size at the time of the first drug administration) was calculated from the actual volumes. To obtain a normal distribution of RTS values, the log of RTS was used. The AUC was calculated and used as the endpoint from the growth curves of the log RTS-values *vs.* time. Such a measure accounts for both degree and duration of inhibition, and the daily fluctuations will be smoothed out in the process, leading to area calculation (Lesser *et al.*, 1980).

2. *Specific growth delay* (*SGD*). SGD was calculated according to Berman & Steel (1984). From the growth curves of the log RTS versus time the time taken for each treated and control tumour to double in volume (DT) was obtained. Tumour growth delay was calculated as the difference between the DT of the individual tumour and the mean DT for control tumours. When this was divided with the mean DT for control tumours the SGD was obtained. SGD can be regarded as the number of DT gained by the treatment. In order to avoid potential pitfalls (Begg, 1980) we analysed growth curves of individual tumours rather than mean of groups.

*Drugs*

The dose of CDDP administered was given to correspond to the maximum tolerated dose (MTD) for tumour-bearing nude mice. At the MTD the mice should have weight loss of 10% in the week following the first injection. Death occurring within two weeks after the last injection was considered a toxic death, and the animal was excluded from evaluation. CDDP and MCA were obtained as commercially available preparations from Bristol Laboratories, Syracuse, NY, USA (Platinol), and H. Lundbeck A/S, Copenhagen, Denmark (Primperan), respectively. The drugs were diluted to proper concentrations before administration intraperitoneally in volumes of 0.01–0.02 ml per g body weight.

The dose of CDDP applied in this study (7.5 mg $kg^{-1}$) was based on results in previously reported dose–response studies (Wennerberg *et al.*, 1984, 1988). In CDDP-sensitive lines it gives a significant inhibition of tumour growth with <10% mortality and <10% weight loss. The MCA dose was calculated from the dose currently being used in humans (Gralla *et al.*, 1981; Tropé *et al.*, 1985).

Two administration schedules were tested: (a) MCA (2.0 mg $kg^{-1}$ i.p.) one hour before CDDP (7.5 mg $kg^{-1}$ i.p.); and (b) MCA (3 x 2.0 mg $kg^{-1}$) given concomitantly, 24 and 48 h after CDDP (7.5 mg $kg^{-1}$) administration. In both schedules the combined treatment was compared with CDDP alone, MCA alone and with NaCl-treated control tumours. The first schedule was tested on tumour line AB and the second on both.

Schedule (a) was first tried. Since MCA given before CDDP did not enhance the effect of CDDP (see **Results**), schedule (b) was applied. The schedule (b) sequence was chosen since it is known that both MCA and CDDP have short half-lifes ($T_{1/2}$ values of 157 and 15 min, respectively: Bateman *et al.*, 1980; Vermorken *et al.*, 1984). In addition, although CDDP has a short $T_{1/2}$ it also has profound effects on DNA synthesis and cell cycle phase distribution for 48 h after drug administration (Wennerberg *et al.*, 1984).

*Statistics*

Data analysis was carried out using the RS/1 data analysis system (Bolt, Beranek and Newman Research Systems, MA, USA). Normality of distributions was tested with the Wilk–Shapiro test. Differences within each experiment were tested with one-way analysis of variance (ANOVA) and then between different treatments with Student's *t* test (population normal) or Mann–Whitney *U* test (population not normal). Differences were tested at a significance level of $P = 0.05$.

**Results**

There was no mortality in any treatment group in any schedule. NaCl administration three times caused *per se* a temporary retardation of weight gain, as did MCA alone. CDDP-containing regimens induced up to 8% weight loss, but weight gain was resumed as soon as drug administration was ended (Figure 1).

Tumour growth was evaluated by one-way analysis of variance (ANOVA) for all treatment groups before comparisons between experimental groups were carried out to evaluate MCA enhancement of CDDP antitumour activity. The *P* values are given in Tables I to III. In the two first experiments (Tables I and II) the hypothesis that the samples

**Figure 1** Weight curves showing changes in median values relation to starting weight for control and treated animals in schedule (b) CDDP: cisplatin, MCA: metoclopramide, C+M: CDDP+MCA. (Points are median of 8–10 animals.)

**Table I** Specific growth delay (SGD) and area under the growth curve (AUC) for tumours of tumour line AB treated according to schedule (a)

| *Treatment group* | *n* | *Mean SGD ±s.e.m.* | *Mean AUC ±s.e.m.* |
|---|---|---|---|
| NaCl | 8 | 0.00±0.36 | 19.67±1.47 |
| CDDP | 7 | 2.14±1.02 | 6.64±2.49 |
| MCA | 9 | 0.73±0.54 | 14.61±2.24 |
| CDDP+MCA | 9 | 2.09±0.85 | 7.94±1.50 |
| ANOVA | | $P = 0.115$ | $P = 0.030$ |

**Table II** Specific growth delay (SGD) and area under the growth curve (AUC) for tumours of tumour line AB treated according to schedule (b)

| *Treatment group* | *n* | *Mean SGD ±s.e.m.* | *Mean AUC ±s.e.m.* |
|---|---|---|---|
| NaCl | 8 | 0.00±0.21 | 16.2±1.15 |
| CDDP | 6 | 0.68±0.40 | 12.1±2.14 |
| MCA | 5 | −0.05±0.26 | 16.2±1.61 |
| CDDP+MCA | 7 | 1.31±0.33 | 5.5±0.44 |
| ANOVA | | $P = 0.012$ | $P = 0.0001$ |

**Table III** Specific growth delay (SGD) and area under the growth curve (AUC) for tumours of tumour line EH treated according to schedule (b)

| *Treatment group* | *n* | *Mean SGD ±s.e.m.* | *Mean AUC ±s.e.m.* |
|---|---|---|---|
| NaCl | 9 | 0.00±0.18 | 10.90±1.14 |
| CDDP | 10 | 0.54±0.18 | 8.58±1.00 |
| MCA | 8 | 0.11±0.24 | 9.71±0.95 |
| CDDP+MCA | 10 | 1.40±0.33 | 4.76±1.00 |
| ANOVA | | $P = 0.001$ | $P = 0.001$ |

came from populations with equal means could be rejected only on the basis of AUC-values as the endpoint. The distribution of AUC-values was normal for all groups, while the distribution of SGD-values in some cases was not normal and non-parametric tests had to be applied.

CDDP alone induced a significant inhibition of tumour growth in tumour line AB in the first experiment with schedule (a) (Table I) (SGD: $P<0.05$; AUC: $P=0.001$) and a close to significant (SGD: 0.13; AUC: $P=0.08$) inhibition in the second experiment with schedule (b) (Table II, Figure 2). In tumour line EH CDDP did not induce any growth retardation of significance (Table III, Figures 3 and 4).

MCA alone did not induce any significant reduction in AUC regardless of treatment, schedule or tumour line used. The addition of MCA to the CDDP treatment did not give any significant additive effect with schedule (a). However, using schedule (b) MCA potentiated the effect of CDDP for both tumour line AB (SGD: $P=0.245$; AUC: $P=0.029$) and EH (SGD: $P=0.047$; AUC: $P=0.015$). In tumour line AB CDDP alone reduced AUC to 75% of control tumours whereas CDDP+MCA reduced AUC to 34% of control tumours (Figure 2). The corresponding values for tumour line EH were 79 and 44%, respectively (Figure 4).

**Figure 2** Area under the growth curve values for control and treated tumours of tumour line AB in schedule (b) as percentage of NaCl-treated controls. CDDP: cisplatin, MCA: metoclopramide, C+M: CDDP+MCA. (Bars are mean of 5–8 tumours ±s.e.m.)

**Figure 3** Growth curves of log RTS (relative tumour size) for tumour line EH in schedule (b). CDDP: cisplatin, MCA: metoclopramide, C+M: CDDP+MCA. (Points are mean of 8–10 tumours ±s.e.m.)

**Figure 4** Area under the growth curve values, tumour weight day 21 and calculated volume day 21 for control and treated tumours of tumour line EH in schedule (b) as percentage of NaCl-treated controls. CDDP: cisplatin, MCA: metoclopramide, C+M: CDDP+MCA. (Bars are mean of 8–10 tumours ±s.e.m.)

The tumours of tumour line EH were excised and weighed after the last measurement on day 21. The correlation coefficient between calculated tumour volume and measured weight was 0.939 ($n=37$) and there was a strong correlation between calculated volume and actual tumour weight down to at least a volume of 145 mm$^3$ (diameter 6.5 mm). Neither weight nor calculated tumour volumes at day 21 of the four treatment groups was as sensitive as SGD and AUC to detect induced growth inhibition (Figure 4).

### Discussion

The present investigation demonstrates that MCA, a non-cytotoxic anti-emetic drug, at the doses used in the present study enhances the effect of CDDP without any increase in mortality or weight loss in the xenograft tumour model system. These effects were observed at a MCA dose comparable to the dose currently being administered to humans (Gralla *et al.*, 1981; Tropé *et al.*, 1985). This interaction has to our knowledge not been described previously.

The rationale for using xenografted human tumours is that they retain their sensitivity to chemotherapeutic drugs. The relevance of this is reflected in findings that the sensitivity of xenografted tumours to commonly used drugs correlates to clinical experience of tumours of the same histological type (Giovanella *et al.*, 1978, 1983). There is also accumulating evidence of a similar response in direct patient/tumour comparisons (Fodstad *et al.*, 1980; Fujita *et al.*, 1980; Shorthouse *et al.*, 1980; Tropé & Wennerberg, 1985).

We have used perpendicular diameters to calculate tumour volume in the evaluation of the MCA-enhancing antitumour properties of CDDP. The accuracy of this method is supported by the present findings, where $r=0.939$ between tumour volume and tumour weight was calculated. The area under the curve (AUC), one of two endpoints used in the present study, is superior to, for example, treatment/control (T/C) ratio in measuring growth inhibition, since AUC accounts for both degree and duration of growth-inhibition. This was demonstrated in the present study when AUC estimations, but not tumour weight or volume at day 21, detected the initial, transient retardation of growth induced by CDDP (Figures 3 and 4). In the xenograft model AUC also seemed to be more sensitive than SGD (Tables I to III).

Benzamide and its analogue are known to be effective sensitisers of ionising radiation and chemotherapeutic drugs, which is presumably modulated via their inhibitory properties of ADPRT (see Introduction for review). A crucial structural feature for effective ADPRT inhibition was the presence of a ring-carboxamide group (Sims *et al.*, 1982), but even when the carboxamide group is polysubstituted with different functional groups, the properties of sensitisation of benzamide analogues were not lost. Whether the sensitising properties of metoclopramide can be related directly to ADPRT modulation, as has been the case with other benzamide analogues, is not known, but it is currently under investigation in our laboratory.

Clinical trials are now in progress to assess the effect of CDDP in patients with squamous cell carcinoma of the head and neck (Morton *et al.*, 1985; Jacobs *et al.*, 1986). The present finding that MCA can enhance the effect of CDDP without any increase in toxicity, at least in an animal model, and the fact that MCA is one of several alternate drugs that can be chosen to control CDDP-induced nausea, suggest important therapeutic possibilities and clearly indicate the relevant aspects of the present study for evaluation in humans.

This investigation was supported by grants from the Swedish Cancer Society (88:156, 88:370), the King Gustaf V Jubilee Fund (87:535), the John and Augusta Persson Foundation for Scientific Medical Research, the Inga Britt and Arne Lundberg Research Foundation, the Funds of the Medical Faculty, University of Lund and foundations of Lund's Health District Organisation.

### References

BATEMAN, D.N., KAHN, C. & DAVIES, D.S. (1980). The pharmacokinetics of metoclopramide in man with observations in the dog. *Br. J. Pharmacol.*, **9**, 371.

BEGG, A.C. (1980). Analysis of growth delay data: Potential pitfalls. *Br. J. Cancer*, **41**, suppl. IV, 93.

BEN-HUR, E. (1984). Involvement of poly (ADP-ribose) in the radiation response of mammalian cells. *Int. J. Radiat. Biol.*, **46**, 659.

BERGER, N.A. (1986). Cancer chemotherapy: New strategies for success. *J. Clin. Invest.*, **78**, 1131.

BERMAN, R. & STEEL, G.G. (1984). Induced and inherent resistance to alkylating agents in human small-cell bronchial carcinoma xenografts. *Br. J. Cancer*, **49**, 431.

FODSTAD, O., AASS, N. & PIHL, A. (1980). Assessment of tumor growth and response to chemotherapy of human melanomas in athymic, nude mice. *Br. J. Cancer*, **41**, suppl. IV, 146.

FUJITA, M., HAYATA, S. & TAGUCHI, T. (1980). Relationship of chemotherapy on human cancer xenografts in nude mice to clinical response in donor patient. *J. Surg. Oncol.*, **15**, 211.

GEORGE, A.M., LUNEC, J., CRAMP, W.A., BRENNAN, S., LEWIS, P.D. & WHISH, W.J.D. (1986). The effects of benzamide ADP-ribosyltransferase inhibitors on the cell survival and DNA strand-break repair in irradiated mammalian cells. *Int. J. Radiat. Biol.*, **49**, 783.

GIOVANELLA, B.C., STEHLIN, J.S., FOGH, J. & SHARKEY, F.E. (1978). Serial transplantation of human malignant tumors in nude mice and their use in experimental chemotherapy. In *Proceedings of the Symposiums on the Use of Athymic (Nude) Mice in Cancer Research*, Houchens, D.P. & Ovejera, A.A. (eds) p. 163. Fischer Verlag: New York/Stuttgart.

GIOVANELLA, B.C., STEHLIN, J.S., SHEPARD, R.C. & WILLIAMS, L.J. (1983). Correlation between response to chemotherapy of human tumours in patients and in nude mice. *Cancer*, **52**, 1146.

GRALLA, R.J., IFRI, L.M., PISKO, S.E. & 6 others (1981). Antiemetic efficacy of high-dose metoclopramide randomized trials with placebo and prochlorperazine in patients with chemotherapy-induced nausea and vomiting. *N. Engl. J. Med.*, **305**, 905.

HORSMAN, M.R., BROWN, D.M., LEMMON, M.J., BROWN, J.M. & LEE, W.W. (1986).Preferential tumor radiosensitization by analogs of nicotinamide and benzamide. *Int. J. Radiat. Oncol. Biol. Phys.*, **12**, 1307.

JACOBS, J.R., KISH, J., ENSLEY, J.F. & 4 others (1986). Combined modality therapy utilizing a cisplatin combination for effective chemotherapy in patients with previously untreated head and neck cancer. *Am. J. Surg.*, **152**, 451.

KJELLÉN, E., PERO, R.W., CAMERON, R. & RANSTAM, J. (1986). Radiosensitizing effects of nicotinamide on a C3H mouse mammary adenocarcinoma. A study on *per os* drug administration. *Acta Radiol.*, **25**, 281.

LESSER, M.L., BRAUN, H.I. & HELSON, L. (1980). Statistical methods for measuring and comparing treatment efficacies: Application to nude mice experimentation. *Exp. Cell Biol.*, **48**, 126.

MORTON, R.P., RUGMAN, F., DORMAN, E.B. & 5 others (1985). Cisplatinum and bleomycin for advanced or recurrent squamous cell carcinoma of the head and neck: a randomised factorial phase III controlled trial. *Cancer Chemother. Pharmacol.*, **15**, 283.

NDUKA, N., SKIDMORE, C.J. & SHALL, S. (1980). The enhancement of cytotoxicity of *N*-methyl-*N*-nitrosurea and of gamma-radiation by inhibitors of poly(ADP-ribose) polymerase. *Eur. J. Biochem.*, **105**, 525.

OSIEKA, R., HOUCHENS, D.P., GOLDIN, A. & JOHNSON, R.K. (1977). Chemotherapy of human colon cancer xenografts in athymic nude mice. *Cancer*, **40**, 2640.

ROSENBERG, B., VANCAMP, L., TROSKO, J.E. & MANSOUR, V.H. (1969). Platinum compounds: A new class of potent antitumour agents. *Nature*, **222**, 385.

SHORTHOUSE, A.J., PECKHAM, M.J., SMYTH, J.F. & STEEL, G.G. (1980). The therapeutic response of bronchial carcinoma xenografts: A direct patient-xenograft comparison. *Br. J. Cancer*, **41**, suppl. IV, 142.

SIMS, J.L., SIKORSKI, G.W., CATINO, D.M., BERGER, S.J. & BERGER, N.A. (1982). Poly(adenosine diphosphoribose) polymerase inhibitors stimulate unscheduled deoxyribonucleic acid synthesis in normal lymphocytes. *Biochemistry*, **21**, 1813.

SKIDMORE, C.J., DAVIES, M.I., GOODWIN, P.M. & 4 others (1979). The involvement of poly(ADP-ribose) polymerase in the degradation of NAD caused by gamma-radiation and *N*-methyl-*N*-nitrosurea. *Eur. J. Biochem.*, **101**, 135.

SMULSON, M.E., SCHEIN, P., MULLINS, D.W. & SUDHAKAR, S. (1977). A putative role for nicotinamide adenine dinucleotide-promoted nuclear protein modification in the antitumor activity of *N*-methyl-*N*-nitrosurea. *Cancer Res.*, **37**, 3006.

SPANG-THOMSEN, M., NIELSEN, A. & VISFELDT, J. (1980). Growth of three human malignant tumours transplanted to nude mice. *Exp. Cell Biol.*, **48**, 138.

THRAVES, P., MOSSMAN, K.L., BRENNAN, T. & DRIFSCHILO, A. (1985). Radiosensitization of human fibroblasts by 3-amino-benzamide, an inhibitor of poly(ADP-ribosylation). *Radiat. Res.*, **104**, 119.

TROPÉ, C., DANNESKIOLD-SAMSOE, P. & HAUKSSON, A. (1985). High-dose metoclopramide in the treatment of *cis*-platinum induced emesis. A dose-finding study. *Neoplasma*, **32**, 507.

TROPÉ, C. & WENNERBERG, J. (1985). Use of tissue culture and nude mice in predictive testing of drug sensitivity in human ovarian cancer of *in vitro* and *in vivo* results. *Abstracts 5th International Workshop on Immuno-deficient Animals, Copenhagen.*

VERMORKEN, J.B., VAN DER VIJGH, W.J.F., KLEIN, I., HART, A.A.M., GALL, H.E. & PINEDO, H.M. (1984). Pharmacokinetics of free and total platinum species after short-term infusion of cisplatin. *Cancer Treat. Rep.*, **68**, 505.

WARD, J.F. (1986). Mechanisms of DNA repair and their potential modification for radiotherapy. *Int. J. Radiat. Oncol. Biol. Phys.*, **12**, 1027.

WARENIUS, H.M., FREEDMAN, L.S. & BLEEHEN, N.M. (1980). The response of a human tumour xenograft to chemotherapy: Intrinsic variations between tumours and its significance in planning experiments. *Br. J. Cancer*, **41**, suppl. IV, 128.

WENNERBERG, J. (1984). Changes in growth pattern of human squamous cell carcinomas of the head and neck during serial passages in nude mice. *Int. J. Cancer*, **33**, 245.

WENNERBERG, J., ALM, P., BIÖRKLUND, A., KILLANDER, D., LÅNGSTRÖM, E. & TROPÉ, C. (1984). Cell cycle perturbations in heterotransplanted squamous cell carcinoma of the head and neck after mitomycin C and cisplatin treatment. *Int. J. Cancer*, **33**, 213.

WENNERBERG, J., BIÖRKLUND, A. & TROPÉ, C. (1988). The effect of cisplatin and 5-fluorouracil on xenografted human squamous cell carcinoma of the head and neck. *Arch. Otolaryngol. Head Neck Surg.*, **114**, 162.